# Risk factors for acute organ failure in intensive care unit patients who receive respiratory support in the absence of non-respiratory organ failure: an international prospective cohort study

**DOI:** 10.1186/cc11306

**Published:** 2012-04-18

**Authors:** Marius Terblanche, Peter Kruger, Stefania di Gangi, Sadiq Gearay, Lynn Gilfeather, Niall D Ferguson, Rupert Pearse, Richard Beale, Andrew Rhodes, Stephen J Brett, Daniel F McAuley

**Affiliations:** 1 Division of Asthma, Allergy Lung Biology, School of Medicine, King's College London, St Thomas Street, London, SE1 9RT, UK; 2 Critical Care & Anaesthesia Research Group, King's Health Partners Academic Health Sciences Centre, Westminster Bridge Road, London, SE1 7EH, UK; 3 Department of Critical Care Medicine, Guy's and St. Thomas' NHS Foundation Trust, Westminster Bridge Road, London, SE1 7EH, UK; 4 Intensive Care Unit, Princess Alexandra Hospital, Ipswich Road, Woolloongabba, Brisbane, QLD 4102, Australia; 5 Department of Anaesthesia and Critical Care, School of Medicine, The University of Queensland, 288 Herston Road, Brisbane, QLD 4006, Australia; 6 Intensive Care Unit, Altnagelvin Hospital, Glenshane Road, Derry, BT47 6SB, Northern Ireland; 7 Interdepartmental Division of Critical Care Medicine, University of Toronto, 200 Elizabeth St., Toronto, ON, M5S 1A8, Canada; 8 School of Medicine, Queen Mary University, Mile End Road, London, E1 4NS, UK; 9 Department of Critical Care Medicine, The Royal London Hospital, Whitechapel Road, London, E1 1BB, UK; 10 Department of Critical Care Medicine, St. George's Healthcare NHS Trust, Blackshaw Road, London, SW17 0QT, UK; 11 Centre for Perioperative Medicine and Critical Care Research, Imperial College Healthcare NHS Trust, Du Cane Road, London, W12 0HS, UK; 12 Centre for Infection and Immunity, Queen's University Belfast, 97 Lisburn Road, Belfast, BT9 7BL, Northern Ireland; 13 Regional Intensive Care Unit, Royal Victoria Hospital, Lisburn Road, Belfast, BT9 7AB, Northern Ireland

## Abstract

**Introduction:**

Many supposed low-risk intensive care unit (ICU) admissions develop acute organ failure (AOF). Identifying patients at high risk of developing AOF and targeting them with preventative strategies may be effective. Our study question was: in a population of ICU patients receiving positive pressure respiratory support (invasive or non-invasive) in the absence of non-respiratory AOF, what is the 14-day incidence of, risk factors for and time to acute organ failure?

**Methods:**

In an international prospective cohort study, patients receiving positive pressure respiratory support (invasive or non-invasive) in the absence of non-respiratory AOF were enrolled and followed for 14 days. The primary outcome measure was the incidence of any AOF (defined as SOFA 3 to 4) during follow-up.

**Results:**

A total of 123 of 766 screened patients (16.1%) were enrolled. Data are reported for 121 patients. In total, 45 out of 121 patients (37.2%) developed AOF. Mortality rates were higher in those with AOF: 17.8% versus 4.0% OR 5.11, *P *= 0.019) for ICU mortality; and 28.9% versus 11.8% (OR 2.80, *P *= 0.019) for hospital mortality. Median ICU length of stay was also longer in those with AOF (11 versus 3.0 days; *P *< 0.0001). Hypoxemic respiratory failure (*P *= 0.001) and cardiovascular dysfunction (that is, SOFA 1 to 2; *P *= 0.03) were associated with AOF. The median time to first AOF was two days.

**Conclusions:**

Patients receiving positive (invasive or non-invasive) pressure respiratory support in the absence of non-respiratory AOF are commonly admitted to ICU; AOF is frequent in these patients. Organ failure developed within a short period after admission. Hypoxemic respiratory failure and cardiovascular dysfunction were strongly associated with AOF.

## Introduction

Patients with severe infections and early sepsis can develop acute organ failure (AOF) [1-3]. Risk of death correlates with the severity and duration of AOF [1]. Except for antibiotics and source control, the current management of sepsis is largely supportive as many potential treatments have failed in randomized trials [4-7]. A different approach to managing AOF is to identify high-risk patients and target them with preventative strategies. For example, Rivers *et al *demonstrated a reduction in mortality when patients with severe sepsis were treated early [8]. However, these patients already suffered AOF. Another example is statin therapy, recently highlighted as a possible efficacious preventative strategy [9,10].

We want to explore whether, in patients with no non-respiratory organ failure who receive positive pressure respiratory support, an opportunity exists to introduce preventative treatments before the onset of multiple organ failure. This approach is based on the premise that mechanical ventilation may be associated with ventilator-associated pneumonia, acute lung injury (ALI) and acute respiratory distress syndrome (ARDS), and also increases the risk of developing other non-respiratory organ failures [11-15].

This international cohort study had three aims. First, to establish whether the target population (at-risk patients receiving positive pressure respiratory support in the absence of non-respiratory organ failure) are admitted to participating ICUs frequently enough to make a randomized controlled trial (RCT) feasible; second, to establish the incidence of AOF and identify baseline risk factors; and third, to confirm the presence of a treatment window sufficiently long to allow the testing of preventative interventions.

We hypothesized that many ICU patients receiving positive pressure ventilatory support develop AOF within 14 days, and that there would be identifiable risk factors for this subgroup. Based on this hypothesis our *a priori *stated study question was: in a population of ICU patients who receive positive pressure respiratory support (invasive or non-invasive) in the absence of non-respiratory organ failure, what is the 14-day incidence of and baseline risk factors for AOF (defined by a sequential organ failure assessment (SOFA) score ≥ 3) [16]?

## Materials and methods

### Study design and setting

We performed an international prospective cohort study. Eleven adult ICUs in three countries participated: nine in the United Kingdom and one each in Canada and Australia. Nine were based in universities and two in general hospitals.

### Participants

Screening took place between May 2009 and March 2010; each centre screened all admissions for an uninterrupted four-week period. All admissions to ICUs were screened during the first 24 hours after intensive care unit/high dependency unit (ICU/HDU) admission. All adults receiving positive pressure respiratory support (invasive or non-invasive) for at least one hour were eligible for inclusion. Positive pressure support included any combination of positive end-expiratory support (PEEP) and positive pressure inspiratory support.

Patients were excluded if they, at the time of meeting the inclusion criteria, had any non-respiratory AOF (defined by a SOFA score ≥ 3 in that organ system) [16]. Moribund patients or those for whom care was limited were also excluded. We excluded elective surgical patients if they were extubated and ready to return to the ward on the morning after admission. To ensure only patients free of non-respiratory AOF were enrolled, we excluded those who, at screening, had any missing data related to the inclusion and/or exclusion criteria.

### Data collection and follow-up

Comprehensive baseline demographic, severity of illness and admission data were entered into a custom-designed database (Microsoft Access™, Microsoft Corp, Seattle, WA, USA). Microbiological results for samples obtained within 48 hours prior to ICU admission were recorded. Daily organ function, physiological, laboratory and treatment data were recorded for up to 14 days. Location on day 28 and vital status at ICU and hospital discharge were recorded.

### Primary outcome

The primary outcome was the incidence of AOF during the follow-up period. AOF, defined as a SOFA ≥ 3, was a composite of any non-respiratory organ failure, or the onset of respiratory failure in only those without respiratory failure (SOFA_resp _< 3) at inclusion [16]. The neurological component was not considered in the analysis.

### Sample size calculations and statistical methods

Data for the screened cohort are presented using values recorded at the time of screening. Baseline data for the eligible cohort are presented using values recorded at the end of the 24-hour screening window. The distributions of all variables were tested for normality; parametric tests were used for with a normal distribution and non-parametric tests for those without. Data are presented as means (standard deviation, SD), median (interquartile range, IQR) and number (percentage, %). Baseline differences between outcome groups were compared using standard tests for continuous and binary variables. We report the number and/or proportion of patients with missing values. Stata/SE Version 11.0 (StataCorp LP, College Station, TX, USA) was used for all analyses. Given the exploratory nature we did not perform an *a priori *sample size calculation. A *P *≤ 0.05 was considered statistically significant for two-sided tests.

Variables associated with AOF (*P *< 0.20) or those potentially associated on clinical or biological grounds were included in regression models. Single and multiple variable models used combinations of χ^2 ^tests and logistic regression techniques that treated AOF as dependent variable.

The study's exploratory nature required non-parsimonious multivariable regression models to identify variables for further exploration in future studies. Models were constructed using automatic stepwise selection estimation with likelihood ratio testing (*P*-value ≤ 0.20) specified as the test of significance to include or exclude variables.

### Ethics approval

For UK sites: the Guy's and St. Thomas' NHS Foundation Trust Multi-Centre Research Ethics Committee (09/H0802/23); for Australia: the Princess Alexandra Hospital Human Research Ethics Committee (2009/029); and for Canada: the University Health Network Research Ethics Board (09-0811-AE). Due to the observational design all three research ethics committees (RECs) waived the need for consent.

## Results

### Admission frequency and characteristics of target population

#### Frequency

**Figure 1:**
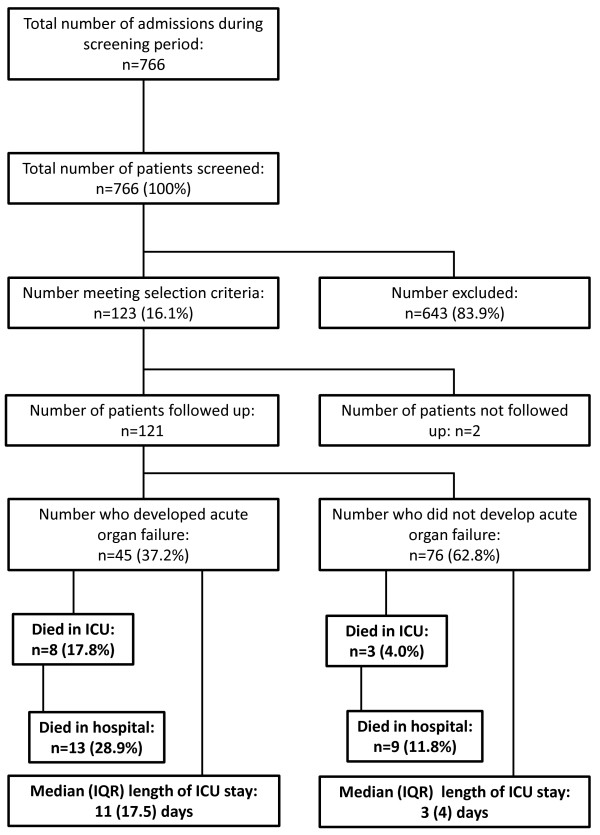
**Flow diagram of study patients and outcomes**.

A total of 766 patients were screened (baseline data presented in Table S1 in Additional file 1); 123 (16.1%) met the inclusion criteria and were eligible for follow-up (Figure 1). Overall mean (SD) age was 57.5 (18) years and the acute physiology and chronic health evaluation (APACHE II) score 14.8 (7.2) for screened patients. Median (IQR) SOFA total score was 4 (6).

No differences in APACHE II (*P *= 0.196) and age (*P *= 0.24) were observed between eligible and non-eligible patients. In non-eligible patients, cardiovascular failure was the most common cause for presentation (*n *= 290/643, 45.1%). Other organ failures present at screening were renal (*n *= 118/643, 18.34%), haematological (*n *= 36/643, 5.6%) and hepatic (*n *= 24/643, 3.7%).

#### Admission characteristics

Two patients met the eligibility criteria but were discharged soon after enrolment; follow-up data are therefore available for 121 patients (Table 1). Mean (SD) age and APACHE II (*n *= 120) were 56.0 (19.1) and 13.8 (6.3), respectively. Most patients had respiratory organ dysfunction or failure at the outset. The median (IQR) respiratory SOFA score was 2 (3). Sixty-three patients (51.2%) had respiratory failure at inclusion. Total SOFA scores including and excluding the respiratory components were 4 (4) and 1 (2), respectively. Additional data not presented here are included in Tables S2 and S4 in Additional file 1.

**Table 1:** Baseline data for eligible patients.

	Cohort, *n *= 121^5^	Progressed to AOF, *n *= 45 (37.2%)	No progression to AOF, *n *= 76 (62.8%)	*P*-value
**Demographic data^1^**				
Age	56.0 (19.1)	58.1 (19.8)	54.7 (18.7)	0.432
Female gender	40 (33.1)	15 (33.3)	25 (32.9)	1.0
APACHE II	13.8 (6.3), *n *= 120	15.8 (5.6)	12.5 (6.2), *n *= 75	0.005
Chronic comorbidities	53 (43.8)	22 (49.0)	31 (40.8)	0.45
Current smoker	23 (19.0), *n *= 83	12 (26.7), *n *= 34	11 (14.5), *n *= 36	0.19
Previous statin therapy	26 (21.5), *n *= 105	8 (17.8), *n *= 36	18 (23.7), *n *= 69	0.23

**Admission characteristics^2^**				
*Reason for admission:*				
Respiratory infection	21 (17.4)	10 (22.2)	11 (14.5)	0.277
COPD/Asthma	8 (6.6)	2 (4.4)	6 (7.9)	0.709
Respiratory	31 (25.6)	8 (17.8)	23 (30.3)	0.128
Sepsis & septic shock	7 (5.8)	5 (11.1)	2 (2.6)	0.100
Shock (CVS)	8 (6.6)	5 (11.1)	3 (4.0)	0.146
CVS (other)	4 (3.3)	2 (4.4)	2 (2.6)	0.628
Trauma	17 (14.1)	7 (15.6)	10 (13.2)	0.714
Neurological	4 (3.3)	0 (0.0)	4 (5.3)	0.296
Drug overdose	2 (1.7)	0 (0.0)	2 (2.6)	0.529
Gastrointestinal	18 (14.9)	6 (13.3)	12 (15.8)	0.714
Other	1 (0.8)	0 (0.0)	1 (1.3)	1.000
*Admission source*				
OPR/Theatre	43 (35.5)	11 (24.4)	32 (42.1)	0.050
Hospital floor/ward	30 (24.8)	15 (33.3)	15 (19.7)	0.094
ER/A&E	30 (24.8)	10 (22.2)	20 (26.3)	0.614
Other hospital area	5 (4.1)	2 (4.4)	3 (4.0)	1.000
Another hospital	13 (10.7)	7 (15.6)	6 (7.9)	0.188
*Other:*				
Readmission to ICU	12 (9.9)	4 (8.9)	8 (10.5)	1.0
Admitted with any infection	28 (23.1)	15 (33.3)	13 (17.1)	0.041
Admitted with pneumonia^3^	21 (75.0)	12 (80.0)	9 (69.2)	0.67
Positive microbiological cultures^3^	42 (40.4)	17 (43.6)	25 (38.5)	0.606

**Organ function - SOFA score^4^**				
Respiratory	2 (3)	3(3)	2(3)	0.019
Renal	0 (1)	0(1)	0(0)	0.048
Haematological	0(0)	0(1)	0(0)	0.197
Cardiovascular	1(1)	1(3)	1(1)	0.002
Hepatic	0(0)	0(1)	0(0)	0.016
Neurological	2(4)	3(3)	2(3)	0.016
Total SOFA score	4(4)	5(5)	3(3)	< 0.0001
Total SOFA score excluding respiratory	1(2)	3(4)	1(2)	< 0.0001

^1^Mean (SD) or total (%) unless otherwise indicated. ^2^N, (%). ^3^Further data available in the online addendum. ^4^Presented as median and interquartile range. ^5^Two patients were discharged soon after admission. AOF, acute organ failure; APACHE, acute physiology and chronic health evaluation; COPD, chronic obstructive pulmonary disease; CVS, cardiovascular; ER/A&E, emergency room/accident & emergency; ICU, intensive care unit; OPR, operating room; SOFA, sequential organ failure assessment.

### Incidence of and risk factors for acute organ failure

#### Incidence

In total, 45 out of 121 patients (37.2%; 95% confidence index (CI) 28.6 to 46.4%) developed AOF. Some 35 patients developed non-respiratory AOF, while 22 out of 60 patients (36.7%) with respiratory SOFA scores < 3 developed frank respiratory failure.

#### Outcomes associated with acute organ failure

The overall ICU and hospital mortalities were 9.2% and 18.2%, respectively (Table 2). Median (IQR) length of ICU stay (iLOS) was 4.0 (8.0) days. Mortality rates were significantly higher in those who developed AOF: 17.8% versus 4.0% odds ratio (OR) 5.11, 95% CI 1.28 to 20.44, *P *= 0.019) for ICU mortality; and 28.9% versus 11.8% (OR 2.80, 95% CI 1.06 to 7.40, *P *= 0.019) for hospital mortality. iLOS also differed significantly: those who developed AOF had a median (IQR) iLOS of 11 (17.5) days versus 3.0 (4.0) for those who did not (*P *< 0.0001).

**Table 2:** Secondary outcome data.

	Cohort, *n *= 121	Progressed to AOF, *n *= 45 (37%)	No progression to AOF, *n *= 76 (63%)	*P*-value
**Mortality^1^**				
ICU	11 (9.2), *n *= 120	8 (17.8)	3 (4.0), *n *= 75	0.019
Hospital	22 (18.2)	13 (28.9)	9 (11.8)	0.019
				
ICU length of ICU stay, days^2^	4.0 (8.0), *n *= 119	11 (17.5), *n *= 44	3.0 (4.0), *n *= 75	< 0.0001

^1^N, (%). ^2^Median (IQR). AOF, acute organ failure; ICU, intensive care unit; IQR, interquartile range.

#### Baseline differences

There were no between-group differences in age, gender or the presence of comorbidities (*P *> 0.05), but APACHE II scores were significantly lower in those who did not develop AOF (15.8 versus 12.5, *P *= 0.005). Whilst admission with an infection was more likely in those who developed AOF (*P *= 0.041), the presence of pneumonia (*P *= 0.67) or positive microbiological results (*P *= 0.606) were not.

At baseline, differences were observed in individual organ system SOFA scores (median, IQR) between those patients that developed AOF and those that did not. Respiratory SOFA was 3 (3) versus 2 (3), *P *= 0.019; renal: 0 (1) versus 0 (0), *P *= 0.048; cardiovascular: 1 (3) versus 1 (1), *P *= 0.002; and hepatic: 0 (1) versus 0 (0), *P *= 0.016. Furthermore, there were also significant differences in total SOFA score (including and excluding the respiratory component) between the two outcome groups (*P *< 0.0001 for both).

#### Univariate analysis

Variables significantly associated with the development of AOF (OR, 95% CI, *P*-value) included (Table 3): APACHE II score (1.09 per APACHE II point, 1.02 to 1.17, 0.01); admission ratio of partial pressure of arterial oxygen to the fraction of inspired oxygen (PaO2/FiO2 ratio) (0.80 per 1 unit change, 0.58 to 1.00, 0.04); receiving positive end-expiratory pressure (PEEP) and positive pressure inspiratory support (8.95, 1.12 to 71.42, 0.04); admission with an infection (2.42, 1.02 to 5.73, 0.04); the presence of cardiovascular dysfunction (2.80, 1.55 to 5.05, 0.03); renal dysfunction (2.21, 1.10 to 4.45, 0.03), and/or hepatic dysfunction (2.67, 1.11 to 6.39, 0.03).

**Table 3:** Risk factors associated with acute organ failure.

Predictor variable	Univariate analysis			Multivariate analysis (*n *= 108)		
	OR^1^	95% CI	*P*-value	**OR**^1^	**95% CI**^1^	***P*-**value^2, 3^
Age (years)	1.00	0.99 - 1.03	0.339			0.381
APACHE II	1.09	1.02 - 1.17 (*n *= 120)	0.008	1.08	1.00 - 1.17	0.064
Female gender	1.02	0.47 - 2.23	0.960			0.668
OPR/Theatre admission	0.44	0.20 - 1.01	0.052			0.324
Admission PaO2/FiO2 ratio	0.80	0.58 - 1.00 (*n *= 114)	0.038			0.498
Hypoxemic failure	4.56	1.98 - 10.53 (*n *= 120)	< 0.001	**5.63**	**1.95 - 16.26**	**0.001**
Type 2 (ventilatory) failure	0.11	0.01 - 0.86 (*n*= 120)	0.035			0.208
Positive end-expiratory pressure support (PEEP) only	0.14	0.02 - 1.12 (*n *= 116)	0.064			0.411
PEEP and positive pressure inspiratory support	8.95	1.12 - 71.42 (*n *= 116)	0.039	7.00	0.61 - 79.79	0.117
Endotracheal tube	2.76	1.16 - 6.59 (*n *= 116)	0.022			0.353
Previous statin therapy	1.10	0.98 - 1.24	0.112	1.16	0.99 - 1.35	0.064
Statin on ICU day 1	0.35	0.09 - 1.32 (*n *= 116)	0.122	0.18	0.03- 1.13	0.068
Admitted with an infection	2.42	1.02 - 5.73	0.044			0.985
Admission lactate	1.06	0.86 - 1.32 (*n *= 110)	0.579			0.209
Admission white cell count	1.00	0.93 - 1.06 (*n *= 115)	0.880			0.570
Cardiovascular dysfunction	2.80	1.55 - 5.05	0.001	**2.06**	**1.02 - 4.14**	**0.044**
Renal dysfunction	2.21	1.10 - 4.45	0.026	2.32	0.93 - 5.79	0.070
Hepatic dysfunction	2.67	1.11 - 6.39	0.028			0.248
Haematological dysfunction	1.86	0.87 - 3.99	0.111			0.714

^1^OR (odds ratio) per one unit change, as appropriate for each variable. ^2^Value adjusted for other variables when OR and 95% CI (confidence interval) presented. ^3^Likelihood ratio value for variable selection when OR and 95% CI not presented. APACHE, acute physiology and chronic health evaluation; ICU, intensive care unit; OPR, operating room; PaO2/FiO2,

#### Multivariable regression analysis

In non-parsimonious multivariable analysis (Table 3) the presence of type 1 respiratory failure (that is, failure of oxygenation; adjusted OR 5.63, 95% CI 1.95 to 16.26, *P *= 0.001) and the presence of cardiovascular dysfunction (that is, SOFA 1 to 2; adjusted OR 2.10, 95% CI 1.07 to 4.12, *P *= 0.03) were associated with AOF. Receiving a statin on the first day of ICU was associated with a trend towards lower risk of AOF (OR 0.18, 95% CI 0.03 to 1.13, *P *= 0.07). Conversely, prior statin therapy (1.16, 0.99 to 1.35, 0.064), the presence of renal dysfunction (2.32, 0.93 to 5.79, 0.07), and APACHE II (1.08 per point, 1.00 to 1.17, 0.06) were associated with a trend towards higher risk.

### Time to acute organ failure

**Figure 2:**
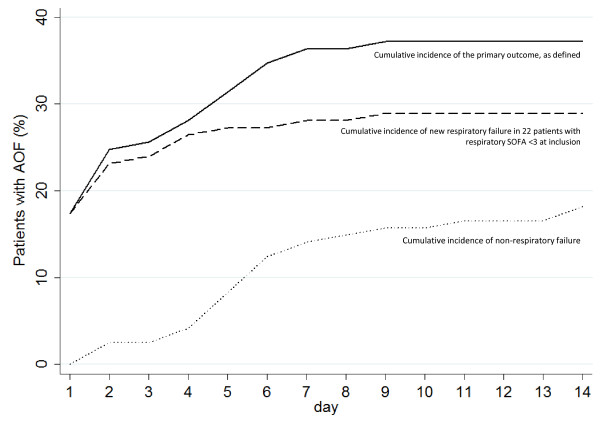
**Cumulative incidence (CInc) of first episode of acute organ failure in any organ system**. Day 1 is the first day of follow-up.

**Figure 3:**
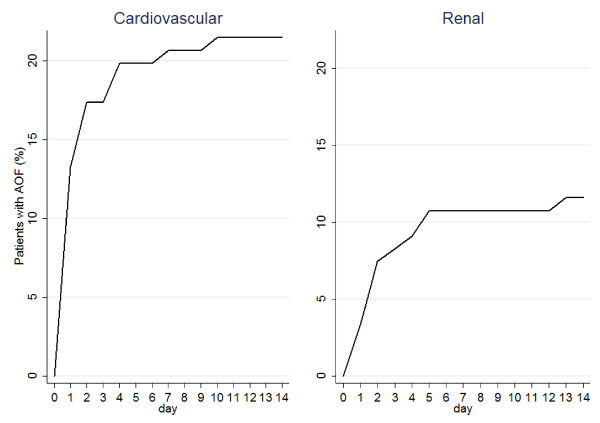
**Cumulative incidence of the first episode of cardiovascular and renal failure**. Day 0 is the day of screening and Day 1 the first day of follow-up. Median time (in days) to acute organ failure is: cardiovascular (1.0), renal (2.0).

The median time to develop AOF was two days (Figure 2). The median time to non-respiratory AOF was one day, contrasting the median six days for respiratory failure to develop in those with a respiratory SOFA score < 3 on admission. The median time to cardiovascular failure was 1.0 day, followed by 1.5 days for haematological and 2.0 days for renal failure (Figure 3).

In those who developed AOF, cardiovascular (*n *= 26/45, 57.8%) and renal (*n *= 14/45, 31.1%) failure were most common (Table S3 in Additional file 1). Interestingly, while few develop haematological or hepatic *failure*, 48.9% and 40.0%, respectively, developed *dysfunction *of these systems.

## Discussion

### Key results

In this international prospective cohort study, nearly one in six (16.1%) admissions met the selection criteria, supporting the supposition that a less sick population is available for enrolment in future trials. The results furthermore show that this population is at high risk (37.2%; 95% CI 28.6 to 46.4%) of AOF, providing key control event rates on which to base future sample size calculations. Failure of oxygenation and the presence of cardiovascular dysfunction were strongly associated with new AOF. Deterioration is associated with important clinical outcomes and resource utilization. Compared to patients who did not develop AOF, those who did were 5.11 (95% CI 1.28 to 20.44) and 2.80 times (95% CI 1.06 to 7.40) more likely to die in ICU and hospital, respectively, and had a median length of stay 4.4 times longer. The median time to AOF was two days. Cardiovascular failure developed within one day, while other organs failed less frequently and over a longer period.

### Interpretation

To our knowledge, this is the first study to investigate specifically and prospectively the risk factors for AOF in a population defined by a therapeutic intervention. We believe this cohort of patients to be easily identifiable, clinically important and at great risk of developing AOF. Identifying modifiable risk factors for AOF and/or testing novel preventative strategies in this population may therefore yield effective interventions.

Epidemiological data on AOF in early critical illness are limited and from different populations. Furthermore, these studies use different definitions for AOF and different scoring systems to assess risks and severity.

Dremsizov *et al *studied hospital patients with community-acquired pneumonia (CAP) and report detailed incidence and time-course data [17,18]. AOF developed in 48% of patients and non-pulmonary organ dysfunction in 39%. Like in our study, renal dysfunction occurred early, but cardiovascular and haematological dysfunction occurred later. The frequently used systemic inflammatory response syndrome (SIRS) criteria were also not associated with organ dysfunction.

Alberti *et al *sought risk factors for worsening sepsis in infected ICU patients admitted to 28 international ICUs [1]. The cumulative incidence of severe sepsis/septic shock was 20% and 24% at days 10 and 30, respectively. Interestingly, ICU mortality ranged from 10.1% in those who did not develop severe sepsis to 95.7% in those who remained in septic shock after 30 days.

Rangel-Fausto *et al *studied 2527 ICU and ward patients with at least two SIRS criteria in a single North American centre [2]. They provided the first evidence that patients with SIRS progressed to sepsis, severe sepsis and septic shock. While numerous infectious and non-infectious illnesses can trigger a systemic inflammatory response, the validity of SIRS criteria is being questioned and available data do not support the use of SIRS criteria, individually or collectively, as a predictive tool [17,19-23].

We recently reported risk factors for AOF in a single-centre retrospective study in which 1397 mechanically ventilated patients without non-respiratory organ failure during the first 24 hours after ICU admission were followed for up to 15 days after admission [24]. APACHE II score and APACHE admission category (cardiovascular and neurological) were strongly associated with AOF.

Our study differs from these studies in two ways. First, we selected patients who, though less severely ill, were still deemed sick enough to receive positive pressure respiratory support. We therefore included all at-risk patients. We believe this to be a major limitation of other work in this area: pre-selection eliminates many patient types and makes it methodologically impossible to study the effect of many predictor variables. For example, including only patients with sepsis makes it challenging to evaluate the effect of an infection on the risk of AOF given that, by definition, all patients should have an infection.

Second, our study used more severe definitions for AOF. Alberti *et al *defined organ dysfunction as a logistic organ dysfunction score (LODS) > 1, while Dremsizov and colleague used previously developed criteria which loosely equates to a SOFA 1 to 2 (although the results remained consistent when a sensitivity analysis using stricter SOFA criteria were performed) [25,26]. The implication is that patients we included with organ dysfunction (that is, with a SOFA 0, 1 to 2) would have been excluded in the other studies, while we used stricter definitions for our primary outcome variable. In effect, we possibly enrolled patients who were already sicker than the other studies, and studied their risk of progressing to a very severe level of sickness.

### Limitations

Our study has a number of limitations. First, the sample size limited our ability to test multiple variables and reduced the precision of the study estimates. Second, the relatively small number of outcome events limited our ability to control for other variables; study estimates are therefore prone to residual confounding. For example, while we believe the multinational recruitment to be a strength, we were unable to assess the effect(s) of clinical practice differences between the UK, Canadian and Australian sites. Third, the short enrollment window at each site may have introduced a selection bias, as the patients admitted during the period may not reflect admissions during the rest of the year. Fourth, the SOFA score categorizes continuous physiological data; using these categories to determine the incidence of a dichotomous outcome may mean that some patients are already in 'biological' organ failure but have not yet crossed the SOFA threshold. This is a limitation inherent in all of the available scoring systems.

## Conclusion

Based on these results, patients who receive positive pressure respiratory support in the absence of non-respiratory AOF are commonly admitted to ICU. This population represent a plausible population for an interventional study of organ protection. Acute organ failure is frequent in these patients and these rates provide key control event rate data. Organ failure developed within a short period after admission but the data confirm the presence of a treatment window. The presence of type 1 (oxygenation) failure and cardiovascular dysfunction are risk factors to consider when future trial selection criteria are designed.

## Key messages

• To improve outcome, interventions aimed at preventing acute organ failure in early critical illness may be better than those used to treat established organ failure

• An at-risk population exists in ICU and this population can be enrolled in future prevention trials.

• Baseline event rates are high, while a treatment window between admission and the development of non-respiratory organ failure appears to exist.

• This study provides crucial data necessary to design future trials.

## Abbreviations

ALI/ARDS: acute lung injury/acute respiratory distress syndrome; AOF: acute organ failure; APACHE: acute physiology and chronic health evaluation; CAP: community-acquired pneumonia; CInc: cumulative incidence; CI: confidence index; COPD: chronic obstructive pulmonary disease; CVS: cardiovascular; ER/A&E: emergency room/accident & emergency; ICU/HDU: intensive care unit/high dependency unit; iLOS: ICU length of stay; IQR: interquartile range; LODS: logistic organ dysfunction score; PaO2/FiO2 ratio: ratio of partial pressure of arterial oxygen to the fraction of inspired oxygen; REC: research ethics committee; OR: odds ratio; OPR: operating room; PEEP: positive end-expiratory pressure; RCT: randomized controlled trial; SD: standard deviation; SIRS: systemic inflammatory response syndrome; SOFA: sequential organ failure assessment.

## Competing interests

The authors declare that they have no competing interests.

## Authors' contributions

MT conceived the study and developed the protocol, participated in the data collection and analysis, and drafted the manuscript. SdG analyzed the data and contributed to the drafting of the manuscript. SG contributed to the study design, managed the data during and after the study, and reviewed the manuscript. PK, SB, AR and NF contributed to the study design, drafting of the manuscript, and revision of the manuscript for important intellectual content. RB, RP and LG contributed to the study design and appraised the manuscript for important intellectual content. DM contributed to the study design, data analysis, drafting of the manuscript, and revision of the manuscript for important intellectual content. All authors read and approved the final version of the manuscript.

MT takes responsibility for the integrity of the work as a whole.

## Supplementary Material

Additional file 1 **Table S1**. Sequential Organ Failure Assessment (SOFA) score. Describes the definitions used for selection criteria and outcome measures. Table S2. Baseline data for all screened patients. Background data of all screened patients, overall and by eligibility. Table S3. Additional day of admission data for eligible patients. Additional admission data, not included in main tables, for patients enrolled in the study. Table S4. Evolution of organ function during follow-up. Evolution of organ function, for individual organ systems, dichotomized by the development of acute organ failure. Click here for file

## Participating sites

The study took place at the following sites (local collaborator).

**Australia: **Princess Alexandra Hospital, Brisbane, Queensland (P Kruger). **Canada: **Toronto General Hospital, Toronto, Ontario (N Ferguson). **United Kingdom: **Altnagelvin Hospital, Derry (L Gilfeather); Antrim Area Hospitals, Antrim (R Bailie); Charing Cross Hospital, London (A Gordon); Guy's and St. Thomas', London (M Terblanche); Hammersmith Hospital, London (S Brett); St. George's Hospital, London (A Rhodes); St. Mary's Hospital, London (R Leonard); The Royal London Hospital, London (R Pearse); and The Royal Victoria Hospital, Belfast (D McAuley).
