# Supplementary material for: Risk factors for acute organ failure in intensive care unit patients who receive respiratory support in the absence of non-respiratory organ failure: an international prospective cohort study
Source: Crit Care. 2012 Apr 18;16(2):R61. doi: 10.1186/cc11306 (PMC3681390; doi:10.1186/cc11306)
Supplement: Additional file 1 — Table S1. Sequential Organ Failure Assessment (SOFA) score. Describes the definitions used for selection criteria and outcome measures. Table S2. Baseline data for all screened patients. Background data of all screened patients, overall and by eligibility. Table S3. Additional day of admission data for eligible patients. Additional admission data, not included in main tables, for patients enrolled in the study. Table S4. Evolution of organ function during follow-up. Evolution of organ function, for individual organ systems, dichotomized by the development of acute organ failure. [file cc11306-S1.DOCX]

**Table S1: Sequential Organ Failure Assessment (SOFA) score**

| **SCORE** | | | | |  |
| --- | --- | --- | --- | --- | --- |
| **ORGAN SYSTEM** | **0** | **1** | **2** | **3** | **4** |
| **Respiratory: PaO_2_/FiO_2_ (mmHg)** | >400 | <400 | <300 | <200 with respiratory support | <100 with respiratory support |
| **Cardiovascular: BP (mmHg)** | MAP>70 without vasopressor | MAP<70 without vasopressor | Dopamine≤5 mcg/kg/min or dobutamine any dose | Dopamine>5 mcg/kg/min or epinephrine≤0.1 mcg/kg/min or norepinephrine≤0.1 mcg/kg/min | Dopamine>15 mcg/kg/min or epinephrine>0.1 mcg/kg/min or norepinephrine>0.1 mcg/kg/min |
| **Renal: creatinine, mg/dL (mmol/L)** | <1.2  (<109) | 1.2-1.9 (110-170) | 2.0-3.4 (171-299) | 3.5-4.9 (300-440) or urine output <500 ml/day | >5.0 (>440) or urine output <200 ml/day |
| **Hematological: (platelet count (x10^3^/mm^3^)** | >150 | <150 | <100 | <50 | <20 |
| **Hepatic: bilirubin, mg/dL (mmol/L)** | <1.2 (<20) | 1.2-1.9 (20-32) | 2.0-5.9 (33-101) | 6.0-11.9 (102-204) | >12.0 (>204) |
| **Glasgow come scale** | 15 | 13-14 | 10-12 | 6-9 | <6 |

From: Vincent JL, Moreno R, Takala J et al. The SOFA (Sepsis-related Organ Failure Assessment) score to describe organ dysfunction/failure. On behalf of the Working Group on Sepsis-Related Problems of the European Society of Intensive Care Medicine. Intensive Care Med 1996;22(7).

**Table S2: Baseline data for all screened patients**

|  | **Overall  (n=766)** | **Eligible for inclusion**  **(n=123, 16%)** | **Not eligible for inclusion**  **(n=643, 84%)** | **p-value** |
| --- | --- | --- | --- | --- |
| APACHE II (mean, SD) | 14.8 (7.2), n=522 | 13.8 (6.3), n=122 | 15.0 (7.5), n=400 | 0.196 |
| Age (mean, SD) | 57.5 (18.0) | 55.8 (19.1) | 57.9 (17.7) | 0.240 |
| **Physiological variables** |  |  |  |  |
| PiO_2_/FiO_2_ ratio, mmHg | 227.5 (171.8), n=719 | 195.8 (140.3), n=122 | 236.3 (171.8), n=597 | 0.0041 |
| Mean blood pressure, mmHg (mean, SD) | 65.7 (15.2), n=762 | 68.66 (13.6) | 65.22 (15.4), n=639 | 0.0115 |
| Creatinine, umol/L | 90.0 (75.0), n=749 | 83.0 (50.00 | 93.0 (87.0), n=626 | 0.0017 |
| 24-hour urine output, ml | 1749.4 (1760.23) | 2133.33 (1648.95) | 1674.55 (1701.18) | <0.0001 |
| Platelets count, x10^9 | 174.0 (120.0), n=750 | 198.0 (123.0) | 170.0 (121.0), n=627 | 0.0173 |
| Bilirubin, umol/L | 14.0 (16.0), n=730 | 13.0 (14.0) | 14.0 (16.0), n=607 | 0.6421 |
| Glascow Come Scale | 15.00 (1.0) | 15.0 (1.0) | 15.0 (2.0) | 0.0129 |
| **SOFA scores** |  |  |  |  |
| Cardiovascular | 1 (3) | 1 (1) | 1 (4) | < 0.0001 |
| Respiratory | 1 (3) | 3 (3) | 1 (3) | 0.0171 |
| Renal | 0 (1) | 0 (1) | 0 (2) | < 0.0001 |
| Haematological | 0 (1) | 0 (1) | 0 (1) | 0.0117 |
| Hepatic | 0 (1) | 0 (1) | 0 (1) | 0.4658 |
| GCS | 0 (1) | 0 (1) | 0 (1) | 0.0116 |
| Total | 4 (6) | 3 (4) | 5 (5) | < 0.0001 |
| **Organ function, N(%)**^1^ |  |  |  |  |
| *Respiratory:* No dysfunction Dysfunction Failure | 370 (48.3) 171 (22.3) 225 (29.4) | 60 (48.8) 0 (0.0) 63 (51.2) | 310 (48.2) 171 (26.6) 162 (25.2) | <0.001 |
| *Cardiovascular:* No dysfunction Dysfunction Failure | 222 (29.0) 254 (33.2) 290 (37.9) | 50 (40.7) 73 (59.4) 0 (0.0) | 172 (26.8) 181 (28.2) 290 (45.1) | <0.001 |
| *Renal:* No dysfunction Dysfunction Failure | 446 (58.2) 202 (26.4) 118 (15.4) | 88 (71.5) 35 (28.5) 0 (0.0) | 358 (55.7) 167 (26.0) 118 (18.4) | <0.001 |
| *Haematological:* No dysfunction Dysfunction Failure | 489 (63.8) 241 (31.5) 36 (4.7) | 91 (74.0) 32 (26.0) 0 (0.0) | 398 (61.9) 209 (32.5) 36 (5.6) | 0.001 |
| *Hepatic:* No dysfunction Dysfunction Failure | 522 (68.2) 220 (28.7) 24 (3.1) | 86 (69.9) 37 (30.1) 0 (0.0) | 436 (67.8) 183 (28.5) 24 (3.7) | 0.057 |
| *Neurological:* No dysfunction Dysfunction Failure | 530 (69.2) 122 (15.9) 114 (14.9) | 92 (74.8) 31 (25.2) 0 (0.0) | 438 (68.1) 91 (14.2) 114 (17.7) | <0.001 |
| **Other variables, N(%)** |  |  |  |  |
| Receiving respiratory support | 518 (67.6) | 123 (100.0) | 395 (61.4) | <0.001 |
| Elective post-operative admission | 234 (30.6) | 20 (16.3) | 214 (33.3) | <0.001 |
| Extubated and ready for discharge on the day of screening | 126 (53.9%) | 0 (0.0) | 126 (58.9) | <0.001 |

Data presented as median and interquartile range, or proportions and percentages unless otherwise stated.

^1^ Dysfunction defined as a SOFA 1-2, and failure as SOFA 3-4.

**Table S3: Additional day of admission data for eligible patients**

|  | **Overall  (n=121)** | **Progressed to AOF (n=45)** | | | | | **No progression to AOF (n=76)** | | **p-value** |
| --- | --- | --- | --- | --- | --- | --- | --- | --- | --- |
| **Physiological and laboratory results** |  |  | | | | |  | |  |
| Lowest temperature^1^ | 36.06(0.89) | 35.96(1.12) | | | | | 36.13(0.71) Missing=5 | | 0.9751 |
| PiO2/FiO2 ratio^1^ | 196.10(93.45) | 173.25(83.70) Missing=0 | | | | | 210.98(97.35) Missing=7 | | 0.0369 |
| Highest lactate^2^ | 1.75(1.20) | 2.10(1.20) Missing=3 | | | | | 1.60(1.15) Missing=8 | | 0.1843 |
| ScvO2^2^ | 71.00(16.50) | 68.00(15.30) Missing=37 | | | | | 72.25(28.55) Missing=64 | | 0.5118 |
| Systolic blood pressure^2^ | 98.00(23.00) | 96.00(30.00) | | | | | 99.00(23.00)  Missing=5 | | 0.4242 |
| Mean arterial blood pressure^1^ | 68.86(12.33) | 68.42(11.86) | | | | | 69.14(12.70)  Missing=6 | | 0.6526 |
| Highest heart rate^1^ | 105.09(21.27) | 107.67(25.47) | | | | | 103.46(18.12) Missing=5 | | 0.3018 |
| Lowest heart rate^2^ | 73.00(21.00) | 77.00(31.00) | | | | | 72.00(19.00)  Missing=5 | | 0.3453 |
| CVP^2^ | 6.00(6.00) | 6.00(4.00)  Missing=12 | | | | | 6.00(6.00)  Missing=38 | | 0.3111 |
| Highest respiratory rate^1^ | 25.37(6.84) | 25.36(6.94) | | | | | 25.38(6.82)  Missing=7 | | 0.9676 |
| Lowest respiratory rate^1^ | 13.10(5.41) | 13.76(5.90) | | | | | 12.69(5.07)  Missing=5 | | 0.2867 |
| WCC^1^ | 12.92(5.84) | 12.82(5.24)  Missing=1 | | | | | 12.99(6.22)  Missing=5 | | 0.5935 |
| Haemoglobin^1^ | 10.20(2.05) | 9.96(2.10) | | | | | 10.35(2.02)  Missing=5 | | 0.1317 |
| Sodium^1^ | 141.36(4.19) | 141.21(5.15)  Missing=3 | | | | | 141.45(3.43) Missing=14 | | 0.2324 |
| AST^2^ | 32.00(117.00) | 90(181)  Missing=25 | | | | | 28(56)  Missing=49 | | 0.0476 |
| ALT^2^ | 30.00(63.00) | 43(81) Missing=9 | | | | | 28(38)  Missing=11 | | 0.2055 |
| C-reactive protein^2^ | 108.00(153.00) | 130(132) Missing=22 | | | | | 93(140) Missing=32 | | 0.1145 |
| Creatinine phosphokinase^2^ | 292.00(685.00) | 536(1473) Missing=39 | | | | | 266(438)  Missing=63 | | 0.1883 |
| Highest blood glucose^2^ | 8.80(4.10) | 9(3) Missing=1 | | | | | 9(4) Missing=5 | | 0.3166 |
| Insulin corresponding to highest glucose^2^ | 0.00(1.00) | | 0(0) Missing=9 | | | | | 0(1)  Missing=14 | 0.2493 |
| Highest insulin dose^2^ | 0.00(3.00) | | 1(3) Missing=13 | | | | | 0(2)  Missing=20 | 0.5786 |
| Glucose corresponding to highest insulin dose^1^ | 7.28(3.37) | | 7.63(2.80) Missing=26 | | | | | 7.08(3.70) Missing=44 | 0.5796 |
| **Types of infection (excluding pneumonia specified elsewhere; N, %)** | | | |  |  |  | | |  |
| Blood stream infection | 1 (3.57%) | | 0 (0.00%) | | | | | 1 (7.69%) | 0.464 |
| Endocarditis | 2 (7.14%) | | 1 (6.67%) | | | | | 1 (7.69%) | 1 |
| CVC | 1 (3.57%) | | 0 (0.00%) | | | | | 1 (7.69%) | 0.464 |
| Peritonitis | 3 (10.71%) | | 2 (13.33%) | | | | | 1 (7.69%) | 1 |
| Urinary trait | 0 (0.00%) | | 0 (0.00%) | | | | | 0 (0.00%) | - |
| Skin and soft tissue | 0 (0.00%) | | 0 (0.00%) | | | | | 0 (0.00%) | - |
| Other (submandibular abscess) | 1 (3.57%) | | 0 (0.00%) | | | | | 1 (7.69%) | 0.464 |
| **Organisms cultured** |  | |  | | | | |  |  |
| MRSA | 1 (2.38%) | | 0 (0.00%)  Missing=2 | | | | | 1 (4.00%) | 1.000 |
| Staph Aureus | 7 (16.67%) | | 4 (23.53%) | | | | | 3 (12.00%) | 0.392 |
| Streptococcus | 2 (4.76%) | | 0 (0.00%) | | | | | 2 (8.00%) | 0.519 |
| E. Coli | 1 (2.38%) | | 0 (0.00%) | | | | | 1 (4.00%) | 1.000 |
| Klebsiella | 2 (4.76%) | | 1 (5.88%) | | | | | 1 (4.00%) | 1.000 |
| Candida (any) | 9 (21.43%) | | 4 (23.53%) | | | | | 5 (20.00%) | 0.705 |
| Fungal (any non-Candida) | 1 (2.38%) | | 1 (5.88%) | | | | | 0 (0.00%) | 0.375 |
| Other | 17 (40.48%) | | 5 (29.41%) | | | | | 12 (48.00%) | 0.364 |
| **Source of organisms cultures** |  | |  | | | | |  |  |
| Non-brochoscopic lavage | 8 (19.05%) | | 5 (29.41%) Missing=2 | | | | | 3 (12.00%) | 0.126 |
| Blood | 2 (4.76%) | | 1 (5.88%) | | | | | 1 (4.00%) | 1.000 |
| Broncho-alveolar lavage | 1 (2.38%) | | 0 (0.00%) | | | | | 1 (4.00%) | 1.000 |
| Urinary tract | 2 (4.76%) | | 0 (0.00%) | | | | | 2 (8.00%) | 0.519 |
| Other | 27 (64.29%) | | 9 (52.94%) | | | | | 18 (72.00%) | 0.433 |
| **Other treatments (N, %)** |  | |  | | | | |  |  |
| Enteral feeding contraindicated | 17 (14.05%) | | 7 (15.56%) | | | | | 10 (13.16%) | 0.714 |
| Receiving enteral feeding | 63 (52.07%) | | 26 (57.78%) | | | | | 37 (48.68%)  Missing=5 | 0.551 |
| Received recombinant activated protein C | 0 (0.00%) | | 0 (0.00%) | | | | | 0 (0.00%) | - |
| Received statin on day 1 | 15 (12.93%) | | 3 (6.67%) Missing=0 | | | | | 12 (15.79%) Missing=5 | 0.157 |
| Use of neuromuscular blocking agent | 6 (4.96%) | | 3 (6.67%) | | | | | 3 (3.95%) Missing=5 | 0.676 |
| Use of sedation | 70 (57.85%) | | 33 (73.33%) | | | | | 37 (48.68%) Missing=5 | 0.023 |
| Steroid administered | 21 (17.36%) | | 7 (15.56%) | | | | | 14 (18.42%) Missing=5 | 0.57 |
| *Type of steroid used* |  | |  | | | | |  |  |
| Hydrocortisone | 7 (33.33%) | | 3 (42.86%) | | | | | 4 (28.57%) | 0.638 |
| Prednisolone | 4 (19.05%) | | 1 (14.29%) | | | | | 3 (21.43%) | 1.000 |
| Methyl-prednisolone | 6 (28.57%) | | 3 (42.86%) | | | | | 3 (21.43%) | 0.354 |
| Dexamethasone | 4 (19.05%) | | 0 (0.00%) | | | | | 4 (28.57%) | 0.255 |
| **Chest X-ray findings (N, %)** |  | |  | | | | |  |  |
| Unilateral infiltrates | 28 (23.14%) | | 9 (20.00%) | | | | | 19 (25.00%) Missing=5 | 0.407 |
| Bilateral infiltrates | 25 (20.66%) | | 13 (28.89%) | | | | | 12 (15.79%) Missing=5 | 0.126 |
| No infiltrates | 37 (30.58%) | | 15 (33.33%) | | | | | 22 (28.95%) Missing=5 | 0.792 |
| Not known | 26 (21.49%) | | 8 (17.78%) | | | | | 18 (23.68%) Missing=5 | 0.34 |
| **Patient location on day 28** |  | |  | | | | |  |  |
| Floor/ward | 31 (25.62%) | | 15 (33.33%) | | | | | 16 (21.05%) | 0.135 |
| Discharged from hospital | 75 (61.98%) | | 20 (44.44%) | | | | | 55 (72.37%) | 0.002 |
| Remains in ICU | 8 (6.61%) | | 6 (13.33%) | | | | | 2 (2.63%) | 0.051 |
| Transferred to another hospital | 7 (5.79%) | | 4 (8.89%) | | | | | 3 (3.95%) | 0.422 |

^1^ Mean (SD)

^2^ Median (IQR)

**Table S4: Evolution of organ function during follow-up^1^**

|  | **Progressed to AOF, n=45 (37%)** | **No progression to AOF, n=76 (63%)** | **p-value** |
| --- | --- | --- | --- |
| **Respiratory:** |  |  |  |
| No dysfunction Dysfunction Failure | 2 (4.4) 4 (8.9) 39 (86.7) | 22 (28.9) 23 (30.3) 31 (40.8) | <0.001 |
| **Cardiovascular:** |  |  |  |
| No dysfunction Dysfunction Failure | 5 (11.1) 14 (31.1) 26 (57.8) | 25 (32.9) 51 (67.1) 0 (0.0) | <0.001 |
| **Renal:** |  |  |  |
| No dysfunction Dysfunction Failure | 20 (44.4) 11 (24.4) 14 (31.1) | 58 (76.3) 18 (23.7) 0 (0.0) | <0.001 |
| **Haematological:** |  |  |  |
| No dysfunction Dysfunction Failure | 19 (42.2) 22 (48.9) 4 (8.9) | 51 (67.1) 25 (32.9) 0 (0.0) | 0.002 |
| **Hepatic:** |  |  |  |
| No dysfunction Dysfunction Failure | 26 (57.8) 18 (40.0) 1 (2.2) | 63 (82.9) 13 (17.1) 0 (0.0) | 0.003 |
| **Neurological:**^2^ |  |  |  |
| No dysfunction Dysfunction Failure | 5(11.1) 7 (15.6) 33 (73.3) | 21 (27.6) 25 (32.9) 30 (39.5) | 0.001 |

^1^ N, (%).

^2^ Neurological score not included in primary outcome variable.

**Online Supplemental Table S5: Baseline respiratory parameters.^1^**

|  | **Cohort, n=121** | **Progressed to AOF n=45 (37%)** | **No progression to AOF n=76 (63%)** | **p-value** |
| --- | --- | --- | --- | --- |
| **Reason for respiratory support,** n=120 |  |  |  |  |
| Routine post-operative | 26 (21.5) | 7 (15.6) | 19 (25.0) | 0.208 |
| Neurological | 15 (12.4) | 3 (6.7) | 12 (15.8) | 0.163 |
| Upper airway | 18 (14.9) | 6 (13.3) | 12 (15.8) | 0.692 |
| Hypoxemic failure | 35 (28.9) | 22 (48.9) | 13 (17.1) | <0.001 |
| Type 2 (ventilatory) failure | 14 (11.6) | 1 (2.2) | 13 (17.1) | 0.016 |
| Trauma | 7 (5.8) | 3 (6.7) | 4 (5.3) | 1.000 |
| Non-respiratory infection | 2 (1.7) | 2 (4.4) | 0 (0.0) | 0.139 |
| Other | 3 (2.5) | 1 (2.2) | 2 (2.6) | 1.000 |
| **Ventilatory mode, n=116** |  |  |  |  |
| None^2^ | 1 (0.8) | 0 (0.0) | 1 (1.3) | 1.000 |
| Positive end-expiratory support (PEEP) only) | 11 (9.1) | 1 (2.2) | 10 (13.2) | 0.048 |
| PEEP and positive-pressure inspiratory support | 103 (85.1) | 44 (97.8) | 59 (77.6) | 0.015 |
| HFOV | 1 (0.8) | 0 (0.0) | 1 (1.3) | 1.000 |
| **Means of ventilator support, n=116** |  |  |  |  |
| None | 8 (6.6) | 0 (0.0) | 8 (10.5) | 0.022 |
| Non-invasive | 21 (17.4) | 8 (17.8) | 13 (17.1) | 0.942 |
| Endotracheal tube | 78 (64.5) | 36 (80.0) | 42 (55.3) | 0.020 |
| Tracheostomy | 9 (7.4) | 1 (2.2) | 8 (10.5) | 0.151 |

^1^ N, (%).

^2^ One patient received positive-pressure support during screening, but did not require support beyond the end of the 24 hour screening window.
